# Rational and design of the digital diagnosis of cardiac sounds in paediatric patients (DI_SOUND) study

**DOI:** 10.1093/ehjdh/ztag103

**Published:** 2026-07-02

**Authors:** Gabriele Egidy Assenza, Alessandro Colombo, Vittoria Mastromarino, Angela D’Aversa, Ylenia Bartolacelli, Simone Bonetti, Giulio Calcagni, Luigi Corvaglia, Luca Di Ienno, Andrea Donti, Maria Francesca Fierro, Matteo Matteucci, Raffaella Marzullo, Carolina Putotto, Maria Giovanna Russo, Giuliana Simonazzi, Sara Bego, Paolo Versacci, Bruno Marino Taussig de Bodonia

**Affiliations:** IRCCS Azienda Ospedaliero-Universitaria di Bologna, Via G. Massarenti 13, 40138 Bologna, Italia; Politecnico di Milano, Milano, Italia; IRCCS Azienda Ospedaliero-Universitaria di Bologna, Via G. Massarenti 13, 40138 Bologna, Italia; Politecnico di Milano, Milano, Italia; IRCCS Azienda Ospedaliero-Universitaria di Bologna, Via G. Massarenti 13, 40138 Bologna, Italia; IRCCS Azienda Ospedaliero-Universitaria di Bologna, Via G. Massarenti 13, 40138 Bologna, Italia; IRCCS Ospedale Pediatrico Bambin Gesù, Roma, Italia; IRCCS Azienda Ospedaliero-Universitaria di Bologna, Via G. Massarenti 13, 40138 Bologna, Italia; Alma Mater Studiorum Università di Bologna, Bologna, Italy; IRCCS Azienda Ospedaliero-Universitaria di Bologna, Via G. Massarenti 13, 40138 Bologna, Italia; IRCCS Azienda Ospedaliero-Universitaria di Bologna, Via G. Massarenti 13, 40138 Bologna, Italia; IRCCS Azienda Ospedaliero-Universitaria di Bologna, Via G. Massarenti 13, 40138 Bologna, Italia; Politecnico di Milano, Milano, Italia; Azienda Ospedaliera Specialistica dei Colli, Ospedale Monaldi, Napoli, Italia; Azienda Ospedaliero-Universitaria Policlinico Umberto I, Roma, Italia; Azienda Ospedaliera Specialistica dei Colli, Ospedale Monaldi, Napoli, Italia; Università Vanvitelli di Napoli; IRCCS Azienda Ospedaliero-Universitaria di Bologna, Via G. Massarenti 13, 40138 Bologna, Italia; Alma Mater Studiorum Università di Bologna, Bologna, Italy; Azienda Ospedaliero-Universitaria Policlinico Umberto I, Roma, Italia; Sapienza Università di Roma, Roma, Italia; Azienda Ospedaliero-Universitaria Policlinico Umberto I, Roma, Italia; Sapienza Università di Roma, Roma, Italia; Azienda Ospedaliero-Universitaria Policlinico Umberto I, Roma, Italia; Sapienza Università di Roma, Roma, Italia

**Keywords:** Congenital heart disease, Screening, Cardiac auscultation, Digital health, Telemedicine

## Abstract

**Aims:**

Congenital heart diseases (CHD) are the most common birth defects in humans. Current clinical newborn screening for CHD has limitations. Digital elaboration of cardiac sounds with diagnostic purposes has been explored in the recent past in adults and older children. We propose to develop a dedicated software for automatic dichotomous clinical classification of heart sounds in newborns.

**Methods and results:**

The Digital Diagnosis of cardiac SOUNDs in paediatric patients (DI_SOUND) study is a multicentre study with two sequential phases: (i) derivation/training and (ii) validation. Each newborn will undergo a digital recording of cardiac sounds (stored as wav file using the Littmann Core Stethoscope) and comprehensive echocardiogram. In the development phase, sound tracings will undergo processing to remove background noise. They will be segmented to generate sound samples of homogeneous suitable tuned duration, and filtered records will be normalized for preventing inhomogeneity in the extracted features. Data analysis will be then implemented based on handcrafted feature extraction. A DI_SOUND classifier algorithm will be developed (**Aim** 1). In the validation phase, consecutive newborns will undergo clinical (standard) screening and digital screening using the DI_SOUND algorithm. Comprehensive echocardiogram will serve as the reference to assess diagnostic performance of the DI_SOUND algorithm (Aim 2). A cost-effective analysis will be used to model marginal cost-effectiveness of digital screening using clinical screening as comparison (Aim 3).

**Conclusion:**

The study will prove if development and validation of a digital classifier of neonatal cardiac sounds is feasible and how this will impact clinical outcomes, patient care, hospital admissions, and screening efficiency.

**ClinicalTrials.gov ID:**

NCT07542509

## Graphical Abstract

**Graphical Abstract:**
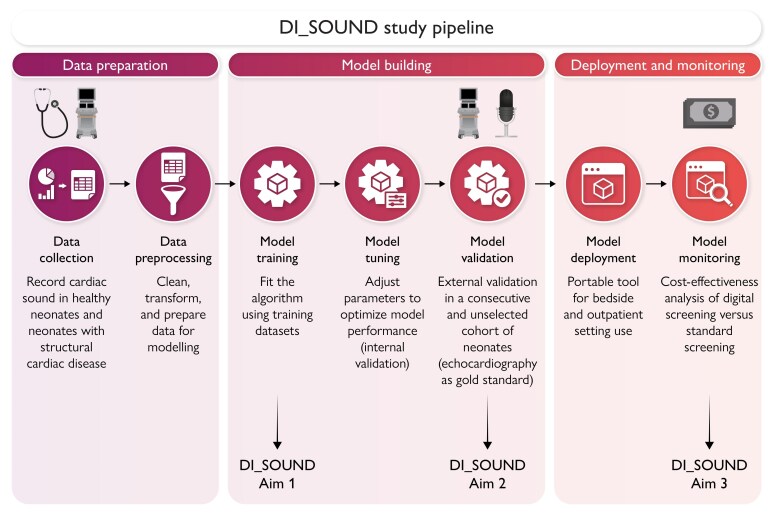
Summary of study pipeline.

## Introduction

Congenital heart diseases (CHD) are the most common birth defects in humans.^1^ One newborn out of one-hundred is born with congenital heart disease.^1^ Moreover, the spectrum of structural heart disease (SHD) in newborns include CHD and acquired (yet with early onset) anomalies such as ventricular dysfunction due to inborn error of metabolism, neonatal cardiomyopathy, myocarditis, extreme prematurity, toxins, arrhythmias, pulmonary disease, and other systemic conditions.^2,3^ Timely diagnosis of SHD in newborns and children is associated with improved outcomes in the general paediatric population.^4^ As example, ductal-dependent CHD are a rare group of cardiovascular malformation with heterogeneous anatomical features, sharing the inability to sustain either the pulmonary (ductal-dependent pulmonary circulation) or the systemic (ductal-dependent systemic circulation) circulation at the time of ductal closure. Examples of such condition are hypoplastic left heart syndrome, severe coarctation of the aorta, pulmonary atresia with intact ventricular septum, critical neonatal aortic valve stenosis, critical pulmonary valve stenosis, and other rare more complex congenital lesions.^5–9^ In this case, it is imperative to timely establish the correct diagnosis to ensure ductal patency through prostaglandin infusion and refer patient for care to tertiary paediatric cardiovascular centres.

Although some SHDs in newborns may present with evident clinical abnormalities (such as cyanosis, heart failure, or low cardiac output), in many cases early manifestations may be overlooked leading to late diagnosis and serious adverse events including neonatal death, making SHD screening in neonates an established field of intervention in public health strategies.^4^ Current newborn screening for SHD predominantly relies on brachial and lower extremity pulse oximetry screening (POS) and cardiac auscultation.^4,10–12^ Diagnostic performance of such practice is limited (see Online Supplementary Material).^13^ POS is plagued by moderate sensitivity, in particular if performed during the first 24 h of life. Cardiac auscultation is probably even more limited with sensitivity ranging between 75–85%.^14–16^ Summary of selected evidence regarding newborn screening (including large observational studies, multicentre implementations, and systematic reviews) are reported in the Online Supplementary Material. The literature consistently shows that POS has moderate sensitivity with detection rates generally ranging from about 70–80% for critical CHD (see Online Supplementary Material). Clinical examination alone, particularly auscultation, has significantly lower sensitivity because many neonates with CHD initially appear asymptomatic and may not have clearly audible murmurs.

Although prenatal and neonatal screening of CHD has been associated with increased recognition of disease in newborns a significant number of patients is not correctly identified and delayed diagnosis is still present in western and even more so in developing countries.^16^

Digital elaboration of cardiac sounds with diagnostic purposes has been explored in the recent past in adults and older children.^17–29^ At this stage of knowledge, no study prospectively derived and validated a digital classifier for cardiac sounds exclusively in newborns (age less than 30 days). Lv *et al*. validated an artificial intelligence assisted auscultation platform based on convolutional neural network in a mixed cohort of about 1300 children [mean age 2.4 years (standard deviation 3.1, median 0.9, and age range 1 day to 15.9 years)] with a sensitivity, specificity and accuracy of 98% [95% confidence interval (CI): 97–99%], 91% (95% CI: 87–95%), and 97% (95% CI: 96–98%), respectively. The *k* coefficient for comparison to face-to-face human auscultation was found to be 0.87 (95% CI: 0.83–0.91).^30^ A similar study was performed by Yang *et al*. in older children using an existing large database of cardiac sounds (restricting the analysis on specific congenital heart defects) with lower diagnostic performance.^31^ But so far there is no commercially available and validated tool for automatic diagnostic screening for SHD in newborns and no such tool has ever received clearance for clinical use.

We propose to develop a dedicated software for automatic screening of heart sounds (normal vs. abnormal) in newborns to improve neonatal recognition of SHD in this population.

The **D**igital d**I**agnosis of cardiac **SOUND** in paediatric patients (DI_SOUND) study aims to develop and validate a tool with the overall goal of improving neonatal recognition of SHD.

## Study aims

Aim 1: Develop a binary classifier for normal vs. abnormal cardiac sounds in newborns.

Aim 2: Validate the binary classifier in a consecutive, independent cohort of newborns.

Aim 3: Cost-effective analysis of digital vs. standard screening modality for CHD in newborns.

## Methods and study design

This is a multicentre study and it will be conducted in four paediatric cardiology programmes in Italy (IRCCS Azienda Ospedaliero-Universitaria di Bologna, IRCCS Ospedale Pediatrico Bambin Gesù in Roma, Azienda Ospedaliero-Universitaria Policlinico Umberto I in Roma and Ospedale Monaldi in Napoli) along with an Engineering unit (Politecnico di Milano). The study is composed of two sequential phases: a derivation/training phase (binary classification algorithm development, Aim 1) and validation phase (binary classification algorithm validation and cost-effective analysis Aims 2 and 3) (*Graphical abstract*). The study design is based on SPIRIT 2025 Guideline. IRB approval has been obtained by each clinical unit.

### Study population

#### Derivation phase

Study population will include neonates with known cardiovascular status including newborns with and without SHD. Working definition of SHD is any cardiovascular anomaly that will require longitudinal follow-up or intervention based on current practice. This broad definition of SHD parallels the overall goal of this investigation: to develop a screening tool aiming for high sensitivity and flexible performance across the diverse and heterogeneous morphological and functional spectrum of cardiovascular anomalies in newborns.

#### Validation phase

Study population will include newborns without previous cardiovascular examination and unknown cardiovascular status. Pre-test probability for SHD will be defined: high-risk newborn for structural cardiovascular abnormalities vs. neonates at low-risk (general population risk level). High-risk sub-group will include newborns with existing foetal ultrasound suggesting cardiovascular abnormalities and those with clinical indication for paediatric cardiology evaluation (abnormal neonatal screening, signs/symptoms). Low-risk sub-group (approaching patient level prevalence for structural cardiovascular abnormalities) will include consecutive neonates specifically enrolled for such research aim and without any indication for cardiovascular examination.

#### Inclusion criteria

- Age < 30 days
- Signed informed consent obtained from parent(s) or representative(s)

#### Exclusion criteria

- Inability to acquire a diagnostic echocardiogram
- Weight less than 1.5 Kg

### Study tool

Cardiac sounds will be recorded using the Littmann Core Stethoscope (Eko Software) (3 M Company, Minnesota/USA). Digital recordings will be stored as wav files in a dedicate encrypted platform for data sharing among centres.

Each newborn enrolled in the study will undergo a standardized and complete echocardiogram by experienced operators as reported in *Table 1* and in the full Study Protocol submitted as Online Supplementary Material. Echocardiographic evaluation will be performed according to existing guidelines for neonatal echocardiography (please refer to the study protocol submitted as Online Supplementary Material for further details).^32^ SHD will be categorized based on the European Society of Cardiology classification system (simple, moderate and severe anatomical complexity), see Supplementary material online, *Table S2*.^33^ Exams will be performed with standardized and reproducible approach (*Table 1*). They will be stored on a digital support. After anonymization, the exams will be transferred to the Echocardiographic Study Core Imaging Laboratory for formal assessment and adjudication.

**Table 1:** Standardized nomenclature, timing, and imaging views for echocardiographic evaluation

Measurement	Timing	View(s)
**TV annulus**	Diastole	Apical 4-chamber
**Pulmonary valve annulus**	Systole	PSAX/PLAX
**Main pulmonary artery**	Systole	PSAX/PLAX
**Left/right pulmonary artery**	Systole	PSAX/PLAX
**Left atrial diameter**	Diastole	PLAX
**MV annulus**	Diastole	PLAX
**MV annulus**	Diastole	Apical 4-chamber
**Aortic root**	Systole	PLAX
**Ascending aorta**	Systole	SSN
**Transverse aortic arc**	Systole	SSN
**Aortic isthmus**	Systole	SSN
**Left ventricular diastolic/systolic area**	Diastole/Systole	Apical 4-chamnber
**Right ventricular diastolic/systolic area**	Diastole/Systole	Modifed apical 4-chamnber
**Left ventricular diameter**	Diastole	PLAX
**Left ventricular diameter**	Systole	PLAX
**TV E wave velocity**	Diastole	Apical 4-chamber
**TV A wave velocity**	Diastole	Apical 4-chamber
**TV deceleration time**	Diastole	Apical 4-chamber
**RV IVRT**	Diastole	Apical 4-chamber
**TV mean gradient**	Diastole	Apical 4-chamber
**TV regurgitant jet velocity**	Diastole	Apical 4-chamber
**RV outflow peak gradient**	Systole	PSAX
**RV outflow mean gradient**	Systole	PSAX
**RV outflow VTI**	Systole	PSAX
**Pulmonary valve peak gradient**	Systole	PSAX
**Pulmonary valve mean gradient**	Systole	PSAX
**Pulmonary valve VTI**	Systole	PSAX
**Left/right branch pulmonary artery peak gradient**	Systole	PSAX
**Left/right branch pulmonary artery mean gradient**	Systole	PSAX
**Left/right branch pulmonary artery VTI**	Systole	PSAX
**MV E wave velocity**	Diastole	Apical 4-chamber
**MV A wave velocity**	Diastole	Apical 4-chamber
**MV deceleration time**	Diastole	Apical 4-chamber
**LV IVRT**	Diastole	Apical 4-chamber
**MV mean gradient**	Diastole	Apical 4-chamber
**MV regurgitant jet velocity**	Diastole	Apical 4-chamber
**MV peak gradient**	Diastole	Apical 4-chamber
**MV mean gradient**	Diastole	Apical 4-chamber
**MV outflow VTI**	Diastole	Apical 4-chamber
**Aortic valve peak gradient**	Systole	Apical 5-chamber
**Aortic mean gradient**	Systole	Apical 5-chamber
**Aortic regurgitant jet velocity**	Systole	Apical 5-chamber
**Aortic valve pressure half-time**	Systole	Apical 5-chamber
**Aortic valve VTI**	Systole	Apical 5-chamber
**Aortic arch peak gradient**	Systole	SSN
**Aortic arch mean gradient**	Systole	SSN
**Aortic arch VTI**	Systole	SSN
**Lateral LV E’ wave TDI**	Diastole	Apical 4-chamber
**Lateral LV A’ wave TDI**	Diastole	Apical 4-chamber
**Septal LV E’ wave TDI**	Diastole	Apical 4-chamber
**Septal LV A’ wave TDI**	Diastole	Apical 4-chamber
**RV free wall E’ wave TDI**	Diastole	Apical 4-chamber
**RV free wall A’ wave TDI**	Diastole	Apical 4-chamber

MV, mitral valve; PLAX, parasternal long AXis; PSAX, parasternal short AXis; RV, right ventricle; SSN, SupraSternal notch; TDI, tissue Doppler imaging; TV, tricuspid valve; VTI, velocity time integral.

A web-based, encrypted Case Report Form will be created using the institutional REDCap (Vanderbilt University) license of IRCCS Azienda Ospedaliero-Universitaria di Bologna.

### Study procedure

#### Patient enrolment

For the derivation phase newborns without SHD will be enrolled at the time of discharge from Ob/Gyn programme and newborns with SHD will be enrolled at the time of cardiovascular examination. For the validation phase consecutive newborns will be enrolled at the time of discharge from Ob/Gyn unit.

*Graphical abstract* summarizes the study pipeline.

#### Derivation phase

After screening of eligible patients, written informed consent will be obtained from parents or caregivers.

The first echocardiographic evaluation will take place at a post-natal age < 30 days. Age at recording and echocardiographic evaluation will be planned not before 7 days of post-natal life to allow completion of proper cardio-circulatory transition in the healthy newborns (i.e. ductal closure, pulmonary vascular remodelling, foramen ovale physiologic shunt). The echocardiogram will be linked to an anonymous identifier which will be used to link the exam to the patient without breaching patient privacy. The examination will be digitally stored and transferred to the Echocardiography Study Core Lab for formal revision. The cardiovascular neonatal status will be appropriately labelled as being with or without cardiovascular abnormalities.

Heart sound will be digitally recorded using as acquisition device Littmann Core Stethoscope (Eko Software) (3 M Company, Minnesota/USA). Cardiac sound tracings will be recorded and stored in wav format and encrypted transferred to the Bioengineer Research Unit for further elaboration. Cardiovascular neonatal status will be un-blinded to the Bioengineer Research Unit to allow for proper handling of classifier training.

The details of the acquisition method are as follows:

1. Acquisition device: Littmann Core Stethoscope (Eko Software) (3 M Company, Minnesota/USA)
2. No filtering of raw signal will be applied. Specifically, the Stethoscope filtering and volume equalization will be set at 0 through the Eko interface. The only residual filter will be the analogue filter related to the stethoscope membrane's elastic properties. This is to minimize the impact of the specificities of the Eko Software on the sound samples and therefore on the training of the classifier.
3. Position of the auscultation: mid precordium, 1 cm medial and 1 cm above papilla mammae (see Online Supplementary Material). Gentle pressure is applied on chest, spontaneous breathing, patients will be kept as calm as possible using maternage without any sedation. Sound sampling will be performed in a calm environment (outpatient clinic or neonatal intensive care unit). A total of three recordings per patient are anticipated to improve sound quality. Sampling tracings with abnormal acoustic contamination (intense crying, environmental noises, friction rubs) will be discarded.
4. Average recording duration: 15 s

We specifically opted for a real-life cardiac auscultation to train the classifier to work in clinically representative setting. The neonate will be handled with maternage and comfortable measures, but no sedation will be used. The clip duration is based on survey data (unpublished) reporting that average neonatal cardiac auscultation in clinic is roughly between 10 and 20 s. Average heart rate in neonate is about 130 beats per minute, as a consequence, 15 s duration clip will record about 30 cardiac cycles. To enhance standardization of recording procedure, we opted for a single recording focus located at the level of mid-precordium (see Figure Online Supplementary Material).

Phonocardiographic signals will be stored as wav files in an encrypted cloud system to be transferred for further analysis (*Graphical abstract*). Tracings will undergo preliminary processing to remove background noise, and will be segmented to generate sound samples of homogeneous suitable tuned duration. As the last step of the preprocessing, the filtered records will be normalized for preventing inhomogeneity in the extracted features. Data analysis will be then implemented based on handcrafted feature extraction. The main objective of feature extraction is to identify a small number of representative features characteristic properties of a sound sample, that replace the high-dimensional raw signals still preserving its informative content with respect to the phenomenon under investigation. Relevant features are then fed to the classification algorithm to discriminate between healthy and abnormal subjects. Multiple different techniques will be used for feature extraction in sound signals, and can be loosely grouped into frequency-domain techniques, time-frequency domain techniques, linear predictive coding, and time-domain techniques. Each of these techniques provides descriptors of potentially relevant properties of the sound sample, which can be used as features or further processed to extract higher level characteristics of the signal (such as systole and diastole variability). To maximize the robustness of our results to less-than-ideal acquisition conditions, we will train the classifiers using features from all the above-mentioned domains and will perform a feature selection procedure to select those features which are mostly correlated to the outcome of interest. Standard machine learning models will be preferred, as simpler models tend to perform more robustly when training data is limited. Neural networks may be implemented if they will exhibit superior results with respect to standard models on the available dataset without clear signs of overfitting. Following the standard best practice, the dataset will be split into training set, validation set and test data set. For evaluating the performance of classifiers, K-Fold cross-validation with different fold numbers will be used: 10-fold, 5-fold, and Leave-One-Out-Cross-Validation (LOOCV). In LOOCV, the number of folds is equal to number of records. Gini importance for Random Forest classifiers will be used as a tool to validate parameters with least predictive contribution. Once a proper classifier has been trained a post training analysis to determine the most relevant features will be conducted. The purpose of this analysis is to investigate to which extent each feature is contributing to the selection of one class with respect to the other. Techniques for feature relevance estimation based on SHAP values, or permutation analysis will be applied also with the aim of providing a proper explanation to the model decision (*Graphical abstract*).

#### Validation phase

Clinical software validation (Aim 2)

After screening of eligible patients written informed consent will be obtained from parents or caregivers.

Clinical screening will be performed as mandated by the Italian law. Pre-ductal (right arm) oxygen saturation and post-ductal (leg) oxygen saturation will be recorded. Physical examination will include femoral arterial pulse detection and face-to-face heart auscultation.

Clinical screening output threshold criteria is summarized in *Table 2*.

**Table 2:** Clinical screening output threshold criteria

	Positive screening	Negative screening
**Oxygen saturation**	<95% and/or arm-to-leg difference >5%	>95% and arm to leg difference < 5%
**Femoral arterial pulse**	Absent or reduced	Normal
**Heart auscultation**	Any divergence from normal cardiac auscultation	Normal I/II heart sound, venous hum, physiologic murmur

For this phase we will enrol patients using a block stratification for high SHD risk vs. low (standard) SHD risk status for each newborn.

Heart recordings will be acquired (15 s length) before comprehensive echocardiography by a trained research investigator. Age at recording and echocardiographic evaluation will be planned not before 7 days of post-natal life to allow completion of proper cardio-circulatory transition in the healthy newborns (i.e. ductal closure, pulmonary vascular remodelling, foramen ovale physiologic shunt).

The echocardiography will be performed according to current guidelines by expert paediatric cardiovascular imager blinded to clinical and digital screening results. Preliminary inter- and intra-observer variability between dedicated expert cardiovascular imagers among centres will be performed in small patient subset.

Pertinent anonymized demographics, cardiovascular status, and pertinent research variables will be stored in an encrypted, web-based, research-focused online software.

Digitally anonymized echocardiographic clips will be transferred to the Echocardiography Study Core Lab who will perform the final, formal reading of the exams dichotomizing patient population into normal and abnormal examination. Any potential conflict of interpretation will be solved with consensus and among all Units cardiac imagers.

**Figure 1:**
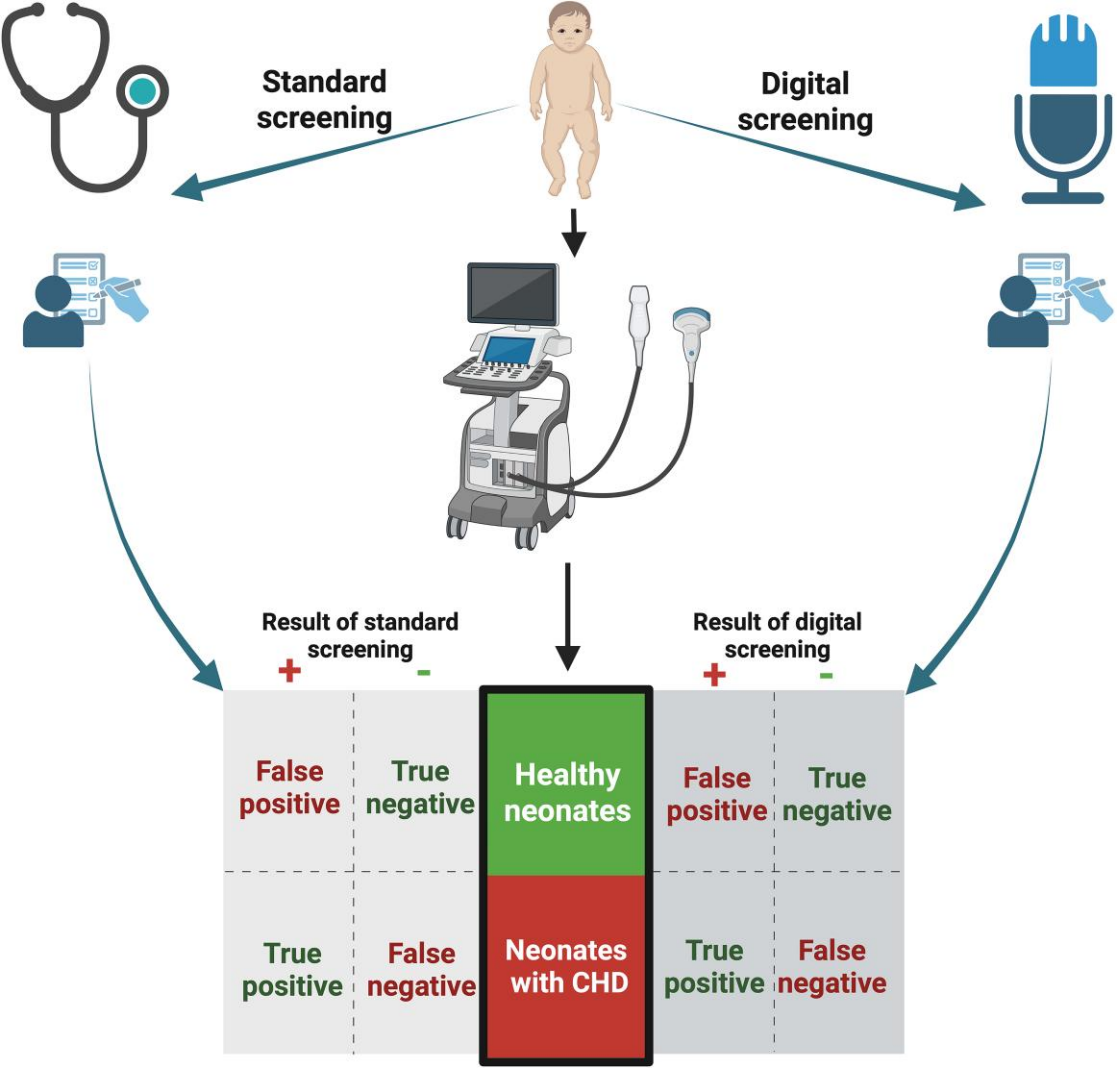
Diagram of validation phase. Each newborn with blinded cardiovascular status will undergo clinical screening as indicated by current practice and regulations and digital screening using the DI_SOUND algorithm. Gold standard evaluation will be comprehensive echocardiography. Pertinent diagnostics are reported at the bottom.

Concordance/discordance pattern between patient-centred comparisons between digital and clinical screening results will be performed using the echocardiography as primary modality of outcome ascertainment (*Figure 1*). False positive will be defined healthy newborns receiving a ‘positive’ screening result using either the clinical (standard or face-to-face auscultation) or the digital approach. Similarly, false negative will be defined newborns with SHD receiving a ‘negative’ screening result using either the clinical (standard or face-to-face auscultation) or the digital approach.

Cost-effectiveness validation (Aim 3)

Aim 3 will evaluate the potential impact of digital screening on healthcare utilization and costs compared with current standard clinical screening. The target population will consist of newborns at low risk for SHD, with no prior indication for cardiovascular examination, in order to approximate the prevalence of undiagnosed cardiovascular abnormalities in the general newborn population.

For the purpose of this analysis, outcomes will be explicitly defined as follows:

- Prevented unnecessary examinations: cases in which (healthy) newborns with a positive result at standard clinical screening but a negative result at digital screening are subsequently confirmed to be free of SHD (i.e. false positives avoided by digital screening).
- Missed necessary examinations: cases in which newborns (with SHD) with a positive result at standard clinical screening but a negative result at digital screening are subsequently diagnosed with SHD (i.e. false negatives introduced by digital screening).

A decision-analytic modelling framework will be developed to compare digital screening with standard clinical practice. Model inputs will include estimates of SHD prevalence, test performance characteristics (sensitivity and specificity of both screening strategies), and downstream diagnostic pathways. Cost estimates will be derived from national diagnosis-related group reimbursement schedules to reflect healthcare system expenditures associated with confirmatory examinations and missed diagnoses.

To account for parameter uncertainty and variability, a probabilistic sensitivity analysis will be conducted using Monte Carlo simulation. This approach will allow repeated sampling from predefined distributions of key parameters (e.g. prevalence, sensitivity, specificity, and costs) to generate distributions of expected costs and outcomes for each screening strategy. The primary outcome will be the incremental cost-effectiveness of digital screening compared with standard clinical screening, expressed in terms of cost per unnecessary examination avoided and cost per missed SHD case.

## Statistical considerations

### Methods of data collection

Patient-level data (demographics, obstetrical history, and family history) will be collected at the time of digital acquisition of heart records, and stored (as previously indicated) in a customary, web-based, properly encrypted database with digital anonymization, by dedicated research personnel. Digital acquisition of heart records will be acquired as previously specified. Echocardiography will be performed by dedicated experienced paediatric cardiovascular imagers. A preliminary inter- and intra-observer variability for the echocardiographic evaluation will be run on 1% of total population (variability testing cohort).

### Statistic plan

Between-group comparisons for clinical and outcome variables will be performed using independent samples *t*-test, Wilcoxon rank sum test, chi-square analysis, or Fisher's exact test using appropriate variable-specific denominators.

For experimental design Aim 1, limited data are available to build up a reliable power calculation at this stage, but we expect to enrol 600 patients with 2:1 diseased:healthy newborn ratio in this experimental phase. The rationale behind the ratio is due to the higher variability of diseased subjects with respect to the healthy ones.

Specific potential classifiers will be studied in the spectral features, cepstral features, and time-domain features. Dataset will be split into training set, validation set, and test data set. The performance of the classifiers will be evaluated using K-Fold cross-validation. Different folding levels will be tested: 10-fold, 5-fold, and LOOCV, aiming for >95% sensitivity and >90% specificity. The numerosity of the sample we target will allow to estimate the target sensitivity and specificity with sufficient accuracy, in particular with the target numerosity we can estimate a 95% confidence interval of ±2% in the sensitivity estimate and ±4% for the specificity estimate.

Gini importance for Random Forest classifiers will be used as a tool to validate parameters with least predictive contribution. Gini importance can be defined as the probability of making a false classification of a randomly chosen record if it were randomly labelled in the class distribution. During the training of the decision trees of the random forest, each tree is a set of internal nodes and leaves. In the internal node, selected feature is used to make decision of dividing the dataset into two separate splits. Selection of the feature is done with Gini impurity criterion. The feature that brings the highest decrease of Gini impurity is selected for the internal nodes. Even after training, how each feature decreases the impurity can be calculated and accepted as the importance of that feature.

Net classification improvement will be used as the major statistical analysis for Experimental Aim 2, using ‘standard of care’ screening modality as comparison group. Sensitivity, specificity, positive, and negative predictive values will be calculated, and likelihood ratio will be analysed. C-statistics will be used to compute diagnostic performance of newly validated algorithm. Bootstrapping will be used to generate confidence intervals.

For experimental design Aim 2, sample size calculation is being presented for sensitivity. Assuming a two-sided α of 0.05% and 90% power, and a disease prevalence of 1% consistent with prior newborn CHD screening studies, we based our calculation on methods for diagnostic test evaluation. Specifically, targeting a sensitivity of approximately 90% with a desired confidence interval width of ±10%, at least 19–20 cases of CHD are required. Given the low expected prevalence (1%), this corresponds to a total sample size of approximately 1900–2000 newborns. This approach is consistent with previously published recommendations for validation of diagnostic algorithms in low-prevalence settings.^34^ Accordingly, an independent validation cohort of approximately 1900 consecutively enrolled participants will be used.

Data will be reported as mean ± standard deviation, median (first and third quartile) or frequency (%). All tests were two-sided. A *P*-value <0.05 was considered significant. Standard statistical analysis will be performed using STATA® 17 h Release data analysis software (StataCorp LP, College Station, TX).

## Discussion

The DI_SOUND study is designed to develop and validate a digital classifier of neonatal cardiac sounds with the aim of improving the efficiency and effectiveness of screening for SHD in newborns. Current neonatal cardiovascular screening strategies rely on pulse oximetry (pre- and post-ductal oxygen saturation) combined with physical examination prior to discharge.^12–16^ While this approach has improved early detection of critical congenital heart disease, its diagnostic performance remains suboptimal, particularly in terms of sensitivity and operator dependency.^16^

Cardiovascular auscultation, historically a cornerstone of neonatal assessment, is inherently subjective and dependent on clinician experience.^14^ Several contemporary factors further limit its effectiveness. First, increasing prenatal detection of SHD has led to centralization of care, reducing exposure of general paediatric trainees and frontline providers to pathological cardiac findings, thereby potentially diminishing auscultatory proficiency.^35^ Second, early postnatal discharge (typically within 48–72 h) may occur before the full haemodynamic expression of certain defects, particularly those involving post-tricuspid shunts, due to persistently elevated pulmonary vascular resistance during transitional circulation. Third, the growing reliance on advanced diagnostic technologies may paradoxically reduce emphasis on meticulous physical examination skills.^35^ Collectively, these factors contribute to the persistence of false-negative screening results, which carry significant clinical risk.

Conversely, current screening strategies are also limited by suboptimal specificity, leading to a considerable number of false-positive cases. This results in unnecessary cardiology referrals, increased healthcare utilization, prolonged hospitalization, and heightened parental anxiety. Therefore, there is a clear unmet need for tools that can enhance both sensitivity and specificity while remaining accessible and scalable.

Within this context, the development of a digital heart sound classifier represents a potentially transformative approach. By leveraging signal processing and machine learning techniques, such a tool may provide objective, reproducible, and operator-independent assessment of cardiac sounds. Importantly, the integration of this technology into widely available digital health and telemedicine platforms could facilitate large-scale deployment, including in low-resource settings where access to specialized neonatal and cardiology expertise is limited.^36^ In such environments, improved early detection of SHD could have a particularly profound impact, as delayed diagnosis is often associated with worse clinical outcomes and limited treatment options.^36^

From a health economics perspective, enhancing early detection of critical, often ductal-dependent, heart defects are highly cost-effective, given the substantial gains in quality-adjusted life years associated with timely intervention—even in resource-constrained healthcare systems.^37^ Thus, improving screening performance is not only clinically relevant but also aligned with broader public health priorities.^38,39^

At the same time, it is important to acknowledge that the implementation of artificial intelligence–based diagnostic tools does not automatically translate into improved clinical outcomes.^40^ Recent evidence suggests that the real-world effectiveness of such technologies depends on multiple factors, including data quality, integration into clinical workflows, user acceptance, and appropriate validation across diverse populations.^40^ Accordingly, the DI_SOUND classifier should be viewed as a complementary tool rather than a replacement for existing screening strategies, with its ultimate value to be determined through rigorous prospective clinical validation. Our ultimate goal is that the DI_SOUND classifier will undergo clinical validation to work as a scalable algorithm for any commercially available telemedicine platform. We believe that a tool easily implementable in commercially available digital health care platforms may have a substantial positive impact in the management of newborns with SHD. Improving screening sensitivity will reduce the number of ‘false negative’ screening results with a potential major impact on patient safety, prognosis, patient care, hospital logistics and this effect may be even more pronounced in low-income setting such as developing countries or remote areas with challenging access to tertiary referral centres, and longer transportation time.^39^ Better screening specificity will reduce the number of ‘false positive’ results with positive effect on health-care cost, reduce family anxiety and newborn discomfort. More importantly, a digital classifier can be easily implemented into tele-medicine web-based platform optimizing screening scalability, penetration, and real-world efficiency.

In summary, this study addresses a clinically significant gap in neonatal cardiovascular screening by proposing an innovative, scalable, and potentially cost-effective solution. If successfully validated, this approach may enhance early detection of SHD, optimize resource utilization, and improve neonatal outcomes, particularly in settings where current screening practices are limited by expertise and infrastructure.

## Limitation

The most notable limitation is the absence of reliable data to calculate power for the development/training phase. Second, some severe SHD (among others transposition of the great arteries with intact ventricular septum) may have minimal acoustic correlates, and, therefore, our approach may be of limited efficiency for these lesions. Third, clinical screening modality integrates a variety of non-acoustic information, such as general newborn appearance, breathing pattern, femoral pulses, oxygen saturation. It is possible that clinical screening method may outperform digital screening for specific subsets of congenital lesions. Fourth, the frequency response of the digital stethoscope may change significantly between stethoscope models, and this may affect the ability of the classifier to transfer performance to a different model. To mitigate this aspect, we have decided to disable all digital filtering and equalization upon sound acquisition. Development and validation of diagnostic tool in a specific world region may constitute *per se* a limit affecting world-wide generalizability. Lastly, small/premature neonates (<1.5 kg) were not included in the target population of our study by design. This neonatal population routinely undergoes comprehensive cardiovascular evaluation, including echocardiographic screening, which reduces the potential added value of a digital heart sound–based screening tool in this subgroup. They frequently present with clinical and physiological conditions (such as mechanical ventilation, underlying lung disease, haemodynamically significant patent ductus arteriosus, and pulmonary vascular abnormalities) that may introduce substantial acoustic variability and confound heart sound recordings. These factors would limit both the reliability of data acquisition and the generalizability of a classifier intended for the broader neonatal population. Fifth, we are aware that a specificity of 90% may be considered lower than expected for a screening test, however, few consideration should be made to this regard: (i) specificity for current clinical screening modality is reported to be between 95% and 99% with a much lower sensitivity (70–80%) and (ii) our boundary condition is assumed under worst case scenario but we aim for a significantly higher specificity and in the Supplementary material online, *Table S3* of the Online Supplementary Material we are reporting a summary table for diagnostic performance of our classifier under different scenarios showing a sustainable workload for lower specificity compared with optimal specificity (about 80 false-positive newborns every/1000). Finally, given the clinical fragility of preterm infants, we carefully considered the ethical implications of additional handling and sensor application and examination, including potential risks of infection and discomfort.

## Conclusion

The DI_SOUND study has the overall aim to transform cardiovascular newborn screening by leveraging technology to enhance diagnostic performance, promote worldwide penetration of telemonitoring screening, reduce health-care cost, improving patient safety. The study will prove if development and validation of a digital classifier of neonatal cardiac sounds is feasible and how this will impact clinical outcomes, patient care, hospital admissions, and screening efficiency.

## Supplementary Material

ztag103_Supplementary_Data

## Data Availability

The data underlying this article are available in the article and in its online supplementary material.

## Lead author biography

Dr. Egidy Assenza is a Consultant Cardiologist and the Adult Congenital Heart Disease Program lead at IRCCS Azienda Ospedaliera Universitaria di Bologna. Dr. Egidy Assenza focused his training in the field of congenital heart disease and adult congenital heart disease cardiology, with a special interest in the evaluation of novel devices and clinical pathways designed for patients with congenital structural heart disease. Dr. Egidy Assenza focused his research on Fontan circulatory failure, Adult Congenital Heart Disease heart failure, pregnancy, and cardiovascular disease.
