# Supplementary material for: Rational and design of the digital diagnosis of cardiac sounds in paediatric patients (DI_SOUND) study
Source: Eur Heart J Digit Health. 2026 Jul 2;7(6):ztag103. doi: 10.1093/ehjdh/ztag103 (PMC13361984; doi:10.1093/ehjdh/ztag103)
Supplement: ztag103_Supplementary_Data [file ztag103_supplementary_data.zip › DI_SOUND_Study_Design_Minor_Revision_Supplementary.docx]

**Table 1 Online Supplementary Material.** Summary Table of selected evidence regarding cardiovascular neonatal screening performance.

| **Study / Evidence Source** | **Study Type** | **Population (N)** | **Screening Method** | **Sensitivity (%)** | **Specificity (%)** | **False Positive Rate** | **Key Findings** |
| --- | --- | --- | --- | --- | --- | --- | --- |
| Cochrane meta-analysis (Plana et al.) | Systematic review / meta-analysis | 436,758 newborns | Pulse oximetry | 76.3 | 99.9 | 0.14% | Detects ~5 of 6 CCHD cases in 10,000 newborns; extremely high specificity (88) |
| Thangaratinam et al. meta-analysis | Systematic review | 229,421 newborns | Pulse oximetry | 76.5 | 99.9 | 0.14% | False-positive rate significantly lower when screening after 24 hours (89) |
| PulseOx Study (Ewer et al.) | Prospective screening study | 20,055 newborns | Pulse oximetry | 75 | 99.2 | 0.84% | Sensitivity lower when antenatal diagnoses excluded (90) |
| POLAR Study | Prospective cohort | 23,959 newborns | Pulse oximetry | 50–70 | 99.1 | ~0.9% | Prenatal detection influences measured screening sensitivity (91) |
| Meta-analysis (Aranguren Bello et al.) | Systematic review | 404,735 newborns | Oximetry + clinical exam | 92 | 98 | ~1% | Combined screening improves detection compared with physical exam alone (92) |

1: Zhang Y, Wang J, Zhao J, Huang G, Liu K, Pan W, Sun L, Li J, Xu W, He C,

Zhang Y, Li S, Zhang H, Zhu J, He Y. Current status and challenges in prenatal

and neonatal screening, diagnosis, and management of congenital heart disease in

China. Lancet Child Adolesc Health. 2023 Jul;7(7):479-489. doi:

10.1016/S2352-4642(23)00051-2. Epub 2023 Jun 7. Erratum in: Lancet Child Adolesc

Health. 2023 Sep;7(9):e17. doi: 10.1016/S2352-4642(23)00176-1. PMID: 37301215.

2: Oster ME, Pinto NM, Pramanik AK, Markowsky A, Schwartz BN, Kemper AR, Hom LA,

Martin GR; and the SECTION ON CARDIOLOGY AND CARDIAC SURGERY; SECTION ON

HOSPITAL MEDICINE; COMMITTEE ON FETUS AND NEWBORN. Newborn Screening for

Critical Congenital Heart Disease: A New Algorithm and Other Updated

Recommendations: Clinical Report. Pediatrics. 2025 Jan 1;155(1):e2024069667.

doi: 10.1542/peds.2024-069667. PMID: 39679594.

3: Ngeow AJ, Tan MG, Choo JT, Tan TH, Tan WC, Chan DK. Screening for congenital

heart disease in a Singapore neonatal unit. Singapore Med J. 2021

Jul;62(7):341-346. doi: 10.11622/smedj.2019167. Epub 2019 Dec 10. PMID:

31820009; PMCID: PMC8801843.

4: Pappas KB. Newborn Screening. Pediatr Clin North Am. 2023

Oct;70(5):1013-1027. doi: 10.1016/j.pcl.2023.06.003. PMID: 37704344.

5: Ford B, Lara S, Park J. Heart Murmurs in Children: Evaluation and Management.

Am Fam Physician. 2022 Mar 1;105(3):250-261. PMID: 35289571.

6: Engvall J. Is it worth screening for congenital structural heart disease in

newborn infants? Acta Paediatr. 2023 Oct;112(10):2028-2029. doi:

10.1111/apa.16903. Epub 2023 Jul 10. PMID: 37431185.

7: Singh Y, Lakshminrusimha S. Perinatal Cardiovascular Physiology and

Recognition of Critical Congenital Heart Defects. Clin Perinatol. 2021

Aug;48(3):573-594. doi: 10.1016/j.clp.2021.05.008. PMID: 34353581.

8: Huang Y, Zhong S, Zhang X, Kong L, Wu W, Yue S, Tian N, Zhu G, Hu A, Xu J,

Zhu H, Sun A, Qin F, Wang Z, Wu S. Large scale application of pulse oximeter and

auscultation in screening of neonatal congenital heart disease. BMC Pediatr.

2022 Aug 12;22(1):483. doi: 10.1186/s12887-022-03540-7. PMID: 35962379; PMCID:

PMC9373434.

9: Oster ME, Kochilas L. Screening for Critical Congenital Heart Disease. Clin

Perinatol. 2016 Mar;43(1):73-80. doi: 10.1016/j.clp.2015.11.005. PMID: 26876122.

10: Hoffman JIE. False negative diagnoses of critical congenital heart disease

with screening neonatal pulse oximetry. J Neonatal Perinatal Med.

2020;13(1):5-9. doi: 10.3233/NPM-190297. PMID: 31594260.

11: Siefkes H, Oliveira LC, Koppel R, Hogan W, Garg M, Manalo E, Cresalia N, Lai

Z, Tancredi D, Lakshminrusimha S, Chuah CN. Machine Learning-Based Critical

Congenital Heart Disease Screening Using Dual-Site Pulse Oximetry Measurements.

J Am Heart Assoc. 2024 Jun 18;13(12):e033786. doi: 10.1161/JAHA.123.033786. Epub

2024 Jun 15. PMID: 38879455; PMCID: PMC11255767.

12: Hamilçıkan Ş, Can E. Critical congenital heart disease screening with a

pulse oximetry in neonates. J Perinat Med. 2018 Feb 23;46(2):203-207. doi:

10.1515/jpm-2017-0006. PMID: 28672762.

13: Cui Y, He XJ, Wang L, Fan YH, Chen JY, Zhao N, Zhang S, Liu L, Yao J, Ren Z,

Fan D, Chen J, He X. A "twelve-section ultrasonic screening and diagnosis

method" and management system for screening and treating neonatal congenital

heart disease at the grassroots level in Tang County, Hebei Province, China. BMC

Pregnancy Childbirth. 2024 May 15;24(1):371. doi: 10.1186/s12884-024-06569-x.

PMID: 38750445; PMCID: PMC11097544.

14: Murni IK, Wibowo T, Arafuri N, Oktaria V, Dinarti LK, Panditatwa D,

Patmasari L, Noormanto N, Nugroho S. Feasibility of screening for critical

congenital heart disease using pulse oximetry in Indonesia. BMC Pediatr. 2022

Jun 27;22(1):369. doi: 10.1186/s12887-022-03404-0. PMID: 35761296; PMCID:

PMC9235153.

15: McClain MR, Hokanson JS, Grazel R, Van Naarden Braun K, Garg LF, Morris MR,

Moline K, Urquhart K, Nance A, Randall H, Sontag MK. Critical Congenital Heart

Disease Newborn Screening Implementation: Lessons Learned. Matern Child Health

J. 2017 Jun;21(6):1240-1249. doi: 10.1007/s10995-017-2273-4. Erratum in: Matern

Child Health J. 2017 Jul;21(7):1590. doi: 10.1007/s10995-017-2295-y. PMID:

28092064; PMCID: PMC5663229.

16: Sasikumar D, Prabhu MA, Kurup R, Francis E, Kumar S, Gangadharan ST,

Mahadevan KK, Sivasankaran S, Kumar RK. Outcomes of neonatal critical congenital

heart disease: results of a prospective registry-based study from South India.

Arch Dis Child. 2023 Nov;108(11):889-894. doi: 10.1136/archdischild-2023-325471.

Epub 2023 Jun 16. PMID: 37328195.

17: Janjua D, Singh J, Agrawal A. Pulse oximetry as a screening test for

congenital heart disease in newborns. J Mother Child. 2022 Jul 20;26(1):1-9.

doi: 10.34763/jmotherandchild.20222601.d-21-00033. PMID: 35853444; PMCID:

PMC10032324.

18: Tsao PC, Chiang SH, Shiau YS, Chen HY, Lin HL, Ho HC, Chen MR, Chang JK,

Wang JK, Chiu SN, Jeng MJ, Hsiao KJ. Comparing Strategies for Critical

Congenital Heart Disease Newborn Screening. Pediatrics. 2023 Mar

1;151(3):e2022057862. doi: 10.1542/peds.2022-057862. PMID: 36815269.

19: Cody F, Franklin O, Mc Cay N, Molphy Z, Dicker P, Breathnach FM. Critical

congenital heart disease: contemporary prenatal screening performance and

outcomes in a multi-centre perinatology service. BMC Pregnancy Childbirth. 2024

Feb 24;24(1):163. doi: 10.1186/s12884-024-06350-0. PMID: 38402176; PMCID:

PMC10893667.

20: Hom LA, Martin GR. Newborn Critical Congenital Heart Disease Screening Using

Pulse Oximetry: Nursing Aspects. Am J Perinatol. 2016 Sep;33(11):1072-5. doi:

10.1055/s-0036-1586108. Epub 2016 Sep 7. PMID: 27603538.

21: Atitlán-Gil A, Mendiola-Figueroa LR, Morales-Argüelles VH, Salomón-Ganado A,

Medécigo-Castelán E, Erdmenger-Orellana J. Implementation of diagnostic

screening for congenital heart disease in Hidalgo, Mexico. Arch Cardiol Mex.

2020;90(1):39-46. English. doi: 10.24875/ACM.19000304. PMID: 31996861.

22: Clausen H, Friberg E, Lannering K, Koivu A, Sairanen M, Mellander M, Liuba

P. Newborn Screening for High-Risk Congenital Heart Disease by Dried Blood Spot

Biomarker Analysis. JAMA Netw Open. 2024 Jun 3;7(6):e2418097. doi:

10.1001/jamanetworkopen.2024.18097. PMID: 38913376; PMCID: PMC11197454.

23: Kumar H, Abbas A, Ewer AK. Newborn Pulse-Oximetry Screening. Clin Perinatol.

2025 Sep;52(3):539-554. doi: 10.1016/j.clp.2025.06.011. Epub 2025 Jul 19. PMID:

40850715.

24: Yang M, Tian Y, Jia P, Ma X, Ge X, Hu X, Zhao Q, Liu F, Jia B, Yan W, Gu Q,

Huang G. Impact of clinical research on public health policy of neonatal

screening for congenital heart disease in China. Chin Med J (Engl). 2022 Jun

5;135(11):1261-1263. doi: 10.1097/CM9.0000000000002031. PMID: 35830175; PMCID:

PMC9433059.

25: Cave AT, Lowenstein SA, McBride C, Michaud J, Madriago EJ, Ronai C. Pulse

Oximetry Screening and Critical Congenital Heart Disease in the State of Oregon.

Clin Pediatr (Phila). 2021 Jun;60(6-7):290-297. doi: 10.1177/00099228211008704.

Epub 2021 Apr 15. PMID: 33855884.

26: Majani N, Chillo P, Slieker MG, Sharau G, Mlawi V, Mongella S, Nkya D,

Khuboja S, Kwesigabo G, Kamuhabwa A, Janabi M, Grobbee D. Newborn Screening for

Critical Congenital Heart Disease in a Low-Resource Setting; Research Protocol

and Preliminary Results of the Tanzania Pulse Oximetry Study. Glob Heart. 2022

May 26;17(1):32. doi: 10.5334/gh.1110. PMID: 35837363; PMCID: PMC9139018.

27: Fernandes N, Short B, Manja V, Lakshminrusimha S. Critical Congenital Heart

Disease Screening in NICU: Need for Revision and Standardization. Am J

Perinatol. 2017 Dec;34(14):1470-1476. doi: 10.1055/s-0037-1603654. Epub 2017 Jun

14. PMID: 28614866.

28: Jullien S. Newborn pulse oximetry screening for critical congenital heart

defects. BMC Pediatr. 2021 Sep 8;21(Suppl 1):305. doi:

10.1186/s12887-021-02520-7. PMID: 34496777; PMCID: PMC8424789.

29: Kemper AR, Lam WKK, Bocchini JA Jr. The Success of State Newborn Screening

Policies for Critical Congenital Heart Disease. JAMA. 2017 Dec

5;318(21):2087-2088. doi: 10.1001/jama.2017.17626. Erratum in: JAMA. 2018 Jan

9;319(2):195. doi: 10.1001/jama.2017.20672. PMID: 29209703.

30: Martin GR, Ewer AK, Gaviglio A, Hom LA, Saarinen A, Sontag M, Burns KM,

Kemper AR, Oster ME. Updated Strategies for Pulse Oximetry Screening for

Critical Congenital Heart Disease. Pediatrics. 2020 Jul;146(1):e20191650. doi:

10.1542/peds.2019-1650. Epub 2020 Jun 4. PMID: 32499387.

31: Desai K, Rabinowitz EJ, Epstein S. Physiologic diagnosis of congenital heart

disease in cyanotic neonates. Curr Opin Pediatr. 2019 Apr;31(2):274-283. doi:

10.1097/MOP.0000000000000742. PMID: 30730315.

32: Banait N, Ward-Platt M, Abu-Harb M, Wyllie J, Miller N, Harigopal S. Pulse

oximetry screening for critical congenital heart disease: a comparative study of

cohorts over 11 years. J Matern Fetal Neonatal Med. 2020 Jun;33(12):2064-2068.

doi: 10.1080/14767058.2018.1538348. Epub 2019 Jan 4. PMID: 30332903.

33: Diaz Kane MM. Pulse Oximetry Screening for Congenital Heart Defects in the

Newborn Nursery: A Review for the General Pediatrician. Pediatr Ann. 2022

Nov;51(11):e411-e413. doi: 10.3928/19382359-20220913-01. Epub 2022 Nov 1. PMID:

36343177.

34: Methlouthi J, Mahdhaoui N, Bellaleh M, Guith A, Zouari D, Ayech H, Nouri S,

Séboui H. Incidence of congenital heart disease in newborns after pulse oximetry

screening introduction. Tunis Med. 2016 Mar;94(3):231-4. PMID: 27575509.

35: Saganski GF, Freire MHS, Santos WMD. Pulse oximetry test for screening

congenital heart diseases: a systematic review. Rev Esc Enferm USP. 2024 Mar

1;57:e20230215. doi: 10.1590/1980-220X-REEUSP-2023-0215en. PMID: 38426937;

PMCID: PMC10906467.

36: Evans WN, Acherman RJ, Ciccolo ML, Lehoux J, Rothman A, Galindo A. Detecting

Critical Congenital Heart Disease in Nevada. World J Pediatr Congenit Heart

Surg. 2019 Nov;10(6):702-706. doi: 10.1177/2150135119873847. PMID: 31701835.

37: Jiang SL, Zhan YJ, Yan P, Yue Y, Tang J. Pulse Oximetry and Perfusion Index

Screening for Congenital Heart Defects: A Systematic Review and Meta-analysis.

Am J Perinatol. 2023 Nov;40(15):1611-1617. doi: 10.1055/s-0042-1748163. Epub

2022 May 17. PMID: 35580627.

38: Ewer AK. Screening for Critical Congenital Heart Defects with Pulse

Oximetry: Medical Aspects. Am J Perinatol. 2016 Sep;33(11):1062-6. doi:

10.1055/s-0036-1586110. Epub 2016 Sep 7. PMID: 27603536.

39: Rasmussen M, Suttner D, Poeltler D, Katheria AC. Use of Pulse Oximetry

Pulsatility Index Screening for Critical Congenital Heart Disease. Am J

Perinatol. 2024 May;41(S 01):e545-e549. doi: 10.1055/a-1904-9389. Epub 2022 Jul

20. PMID: 35858650.

40: Narayen IC, Blom NA, Ewer AK, Vento M, Manzoni P, te Pas AB. Aspects of

pulse oximetry screening for critical congenital heart defects: when, how and

why? Arch Dis Child Fetal Neonatal Ed. 2016 Mar;101(2):F162-7. doi:

10.1136/archdischild-2015-309205. Epub 2015 Sep 14. PMID: 26369369.

41: Suard C, Flori A, Paoli F, Loundou A, Fouilloux V, Sigaudy S, Michel F,

Antomarchi J, Moceri P, Paquis-Flucklinger V, D'Ercole C, Bretelle F. Accuracy

of prenatal screening for congenital heart disease in population: A

retrospective study in Southern France. PLoS One. 2020 Oct 5;15(10):e0239476.

doi: 10.1371/journal.pone.0239476. PMID: 33017437; PMCID: PMC7535055.

42: Lytzen R, Vejlstrup N, Bjerre J, Petersen OB, Leenskjold S, Dodd JK,

Jørgensen FS, Søndergaard L. Mortality and morbidity of major congenital heart

disease related to general prenatal screening for malformations. Int J Cardiol.

2019 Sep 1;290:93-99. doi: 10.1016/j.ijcard.2019.05.017. Epub 2019 May 7. PMID:

31130278.

43: Aranguren Bello HC, Londoño Trujillo D, Troncoso Moreno GA, Dominguez Torres

MT, Taborda Restrepo A, Fonseca A, Sandoval Reyes N, Chamorro CL, Dennis Verano

RJ. Oximetry and neonatal examination for the detection of critical congenital

heart disease: a systematic review and meta-analysis. F1000Res. 2019 Mar

1;8:242. doi: 10.12688/f1000research.17989.1. PMID: 31372214; PMCID: PMC6659768.

44: Schena F, Picciolli I, Agosti M, Zuppa AA, Zuccotti G, Parola L, Pomero G,

Stival G, Markart M, Graziani S, Gagliardi L, Bellan C, La Placa S, Limoli G,

Calzetti G, Guala A, Bonello E, Mosca F; Neonatal Cardiology Study Group of the

Italian Society of Neonatology. Perfusion Index and Pulse Oximetry Screening for

Congenital Heart Defects. J Pediatr. 2017 Apr;183:74-79.e1. doi:

10.1016/j.jpeds.2016.12.076. Epub 2017 Jan 30. PMID: 28153478.

45: Bin-Nun A, Hammerman C, Mimouni FB, Wasserteil N, Kasirer YM. The Saga of

Pulse Oximetry Screening for Critical Congenital Heart Disease in Israel: A

Historical Perspective. Isr Med Assoc J. 2021 Apr;23(4):229-232. PMID: 33899355.

46: Zarouni SA, Mheiri NMA, Blooshi KA, Serkal YA, Preman N, Naqvi SA, Khan Y.

Impact of an electronic medical record-based automated screening program for

critical congenital heart disease: Emirates Health Services, United Arab

Emirates. BMC Med Inform Decis Mak. 2022 Jun 21;22(1):165. doi:

10.1186/s12911-022-01900-y. PMID: 35729549; PMCID: PMC9214992.

47: Singh Y, Chen SE. Impact of pulse oximetry screening to detect congenital

heart defects: 5 years' experience in a UK regional neonatal unit. Eur J

Pediatr. 2022 Feb;181(2):813-821. doi: 10.1007/s00431-021-04275-w. Epub 2021 Oct

7. PMID: 34618229; PMCID: PMC8821483.

48: Diller CL, Kelleman MS, Kupke KG, Quary SC, Kochilas LK, Oster ME. A

Modified Algorithm for Critical Congenital Heart Disease Screening Using Pulse

Oximetry. Pediatrics. 2018 May;141(5):e20174065. doi: 10.1542/peds.2017-4065.

PMID: 29691284.

49: Wong KK, Fournier A, Fruitman DS, Graves L, Human DG, Narvey M, Russell JL.

Canadian Cardiovascular Society/Canadian Pediatric Cardiology Association

Position Statement on Pulse Oximetry Screening in Newborns to Enhance Detection

of Critical Congenital Heart Disease. Can J Cardiol. 2017 Feb;33(2):199-208.

doi: 10.1016/j.cjca.2016.10.006. Epub 2016 Oct 26. PMID: 28043739.

50: Van Naarden Braun K, Grazel R, Koppel R, Lakshminrusimha S, Lohr J, Kumar P,

Govindaswami B, Giuliano M, Cohen M, Spillane N, Jegatheesan P, McClure D,

Hassinger D, Fofah O, Chandra S, Allen D, Axelrod R, Blau J, Hudome S, Assing E,

Garg LF. Evaluation of critical congenital heart defects screening using pulse

oximetry in the neonatal intensive care unit. J Perinatol. 2017

Oct;37(10):1117-1123. doi: 10.1038/jp.2017.105. Epub 2017 Jul 27. PMID:

28749481; PMCID: PMC5633653.

51: Gong A, Guillory C, Creel L, Livingtson JE, McKee-Garrett TM, Fortunov R. A

Multicenter Initiative for Critical Congenital Heart Disease Newborn Screening

in Texas Neonatal Intensive Care Units. Am J Perinatol. 2017 Jul;34(9):839-844.

doi: 10.1055/s-0037-1599053. Epub 2017 Feb 17. PMID: 28212589.

52: Robinson DL, Craig MS, Wells RS, Liesemer KN, Studer MA. Newborn Screening

Pulse Oximetry to Detect Critical Congenital Heart Disease: A Follow-Up Survey

of Current Practice at Army, Navy and Air Force Hospitals. Mil Med. 2019 Dec

1;184(11-12):826-831. doi: 10.1093/milmed/usz116. PMID: 31090912.

53: Hu XJ, Ma XJ, Zhao QM, Yan WL, Ge XL, Jia B, Liu F, Wu L, Ye M, Liang XC,

Zhang J, Gao Y, Zhai XW, Huang GY. Pulse Oximetry and Auscultation for

Congenital Heart Disease Detection. Pediatrics. 2017 Oct;140(4):e20171154. doi:

10.1542/peds.2017-1154. PMID: 28939700.

54: Fernandes N, Lakshminrusimha S. The limitations of pulse oximetry for

critical congenital heart disease screening in the neonatal intensive care

units. Acta Paediatr. 2017 Jun;106(6):1007. doi: 10.1111/apa.13742. Epub 2017

Jan 30. PMID: 28075515.

55: de Araújo JS, Regis CT, Gomes RG, Mourato FA, Mattos SD. Impact of

Telemedicine in the Screening for Congenital Heart Disease in a Center from

Northeast Brazil. J Trop Pediatr. 2016 Dec;62(6):471-476. doi:

10.1093/tropej/fmw033. Epub 2016 Jun 7. PMID: 27273306.

56: Narayen IC, Blom NA, Bourgonje MS, Haak MC, Smit M, Posthumus F, van den

Broek AJ, Havers HM, te Pas AB. Pulse Oximetry Screening for Critical Congenital

Heart Disease after Home Birth and Early Discharge. J Pediatr. 2016

Mar;170:188-92.e1. doi: 10.1016/j.jpeds.2015.12.004. Epub 2015 Dec 31. PMID:

26746119.

57: Zhao QM, Niu C, Liu F, Wu L, Ma XJ, Huang GY. Accuracy of cardiac

auscultation in detection of neonatal congenital heart disease by general

paediatricians. Cardiol Young. 2019 May;29(5):679-683. doi:

10.1017/S1047951119000799. Epub 2019 Apr 23. PMID: 31012400.

58: Kemper AR, Hudak ML. Revisiting the Approach to Newborn Screening for

Critical Congenital Heart Disease. Pediatrics. 2018 May;141(5):e20180576. doi:

10.1542/peds.2018-0576. PMID: 29691283.

59: Oster ME, Aucott SW, Glidewell J, Hackell J, Kochilas L, Martin GR,

Phillippi J, Pinto NM, Saarinen A, Sontag M, Kemper AR. Lessons Learned From

Newborn Screening for Critical Congenital Heart Defects. Pediatrics. 2016

May;137(5):e20154573. doi: 10.1542/peds.2015-4573. Epub 2016 Apr 15. PMID:

27244826; PMCID: PMC5227333.

60: Abouk R, Grosse SD, Ailes EC, Oster ME. Association of US State

Implementation of Newborn Screening Policies for Critical Congenital Heart

Disease With Early Infant Cardiac Deaths. JAMA. 2017 Dec 5;318(21):2111-2118.

doi: 10.1001/jama.2017.17627. Erratum in: JAMA. 2018 Sep 25;320(12):1288. doi:

10.1001/jama.2018.13235. PMID: 29209720; PMCID: PMC5770276.

61: Kondo M, Ohishi A, Baba T, Fujita T, Iijima S. Can echocardiographic

screening in the early days of life detect critical congenital heart disease

among apparently healthy newborns? BMC Pediatr. 2018 Nov 19;18(1):359. doi:

10.1186/s12887-018-1344-z. PMID: 30453920; PMCID: PMC6241044.

62: Abbas A, Ewer AK. New born pulse oximetry screening: A global perspective.

Early Hum Dev. 2021 Nov;162:105457. doi: 10.1016/j.earlhumdev.2021.105457. Epub

2021 Sep 1. PMID: 34548207.

63: Mouledoux J, Guerra S, Ballweg J, Li Y, Walsh W. A novel, more efficient,

staged approach for critical congenital heart disease screening. J Perinatol.

2017 Mar;37(3):288-290. doi: 10.1038/jp.2016.204. Epub 2016 Nov 10. PMID:

27831548; PMCID: PMC5334208.

64: Kurosaki K, Kitano M, Sakaguchi H, Shiraishi I, Iwanaga N, Yoshimatsu J,

Hoashi T, Ichikawa H, Yasuda S. Discrepancy Between Pre- and Postnatal Diagnoses

of Congenital Heart Disease and Impact on Neonatal Clinical Course　- A

Retrospective Study at a Japanese Tertiary Institution. Circ J. 2020 Nov

25;84(12):2275-2285. doi: 10.1253/circj.CJ-20-0316. Epub 2020 Nov 3. PMID:

33148938.

65: Cawsey MJ, Noble S, Cross-Sudworth F, Ewer AK. Feasibility of pulse oximetry

screening for critical congenital heart defects in homebirths. Arch Dis Child

Fetal Neonatal Ed. 2016 Jul;101(4):F349-51. doi:

10.1136/archdischild-2015-309936. Epub 2016 Feb 25. PMID: 26915671.

66: Migowski A, da Costa GTL, Rey HCV. Newborn pulse oximetry screening coverage

in a nationwide complex survey sample: An assessment of a congenital heart

disease early detection program at the regional level in Brazil. Prev Med. 2024

Dec;189:108141. doi: 10.1016/j.ypmed.2024.108141. Epub 2024 Sep 18. PMID:

39303895.

67: Lai Z, Vadlaputi P, Tancredi DJ, Garg M, Koppel RI, Goodman M, Hogan W,

Cresalia N, Juergensen S, Manalo E, Lakshminrusimha S, Chuah CN, Siefkes H.

Enhanced Critical Congenital Cardiac Disease Screening by Combining

Interpretable Machine Learning Algorithms. Annu Int Conf IEEE Eng Med Biol Soc.

2021 Nov;2021:1403-1406. doi: 10.1109/EMBC46164.2021.9630111. PMID: 34891547;

PMCID: PMC8890698.

68: Yao NA, Agyekum A, Adaboh A, Celestin D, Sayeed S. A pilot implementation

study to detect neonatal critical congenital heart disease using pulse oximetry

screening in Accra, Ghana. BMJ Glob Health. 2026 Jan 28;11(1):e022157. doi:

10.1136/bmjgh-2025-022157. PMID: 41605545; PMCID: PMC12853520.

69: Mukerji A, Shafey A, Jain A, Cohen E, Shah PS, Sander B, Shah V. Pulse

oximetry screening for critical congenital heart defects in Ontario, Canada: a

cost-effectiveness analysis. Can J Public Health. 2020 Oct;111(5):804-811. doi:

10.17269/s41997-019-00280-7. Epub 2020 Jan 6. PMID: 31907759; PMCID: PMC7501328.

70: Therrell BL Jr, Padilla CD. Newborn screening in the developing countries.

Curr Opin Pediatr. 2018 Dec;30(6):734-739. doi: 10.1097/MOP.0000000000000683.

PMID: 30124582.

71: Narayen IC, Te Pas AB, Blom NA, van den Akker-van Marle ME. Cost-

effectiveness analysis of pulse oximetry screening for critical congenital heart

defects following homebirth and early discharge. Eur J Pediatr. 2019

Jan;178(1):97-103. doi: 10.1007/s00431-018-3268-x. Epub 2018 Oct 17. PMID:

30334077; PMCID: PMC6311198.

72: Hu XJ, Zhao QM, Ma XJ, Yan WL, Ge XL, Jia B, Liu F, Wu L, Ye M, Huang GY.

Pulse oximetry could significantly enhance the early detection of critical

congenital heart disease in neonatal intensive care units. Acta Paediatr. 2016

Nov;105(11):e499-e505. doi: 10.1111/apa.13553. Epub 2016 Sep 20. PMID: 27540721;

PMCID: PMC5095792.

73: De Bernardo G, Arienzo MR, Barbieri F, Centanni F, Moccia F, Giordano M,

Buonocore G, Perrone S. The Role of Newborn Pulse Oximetry Screening for

Detecting Critical Congenital Heart Defects: A Narrative Review. Curr Pediatr

Rev. 2025;21(3):227-232. doi: 10.2174/0115733963315664241024043935. PMID:

39484759.

74: Song X, Lu Y, Gu Q, Ding H, Shen C, Kong X, Xie T, Ning W, Lu S. An

evaluation of a five-year program for newborn congenital heart disease screening

in Jiangsu Province, 2019-2023. BMC Public Health. 2025 Feb 10;25(1):552. doi:

10.1186/s12889-025-21698-4. PMID: 39930425; PMCID: PMC11812251.

75: Narayen IC, Blom NA, van Geloven N, Blankman EIM, van den Broek AJM, Bruijn

M, Clur SB, van den Dungen FA, Havers HM, van Laerhoven H, Mir SE, Muller MA,

Polak OM, Rammeloo LAJ, Ramnath G, van der Schoor SRD, van Kaam AH, Te Pas AB;

POLAR study group. Accuracy of Pulse Oximetry Screening for Critical Congenital

Heart Defects after Home Birth and Early Postnatal Discharge. J Pediatr. 2018

Jun;197:29-35.e1. doi: 10.1016/j.jpeds.2018.01.039. Epub 2018 Mar 23. PMID:

29580679.

76: Mikrou P, Singh A, Ewer AK. Pulse oximetry screening for critical congenital

heart defects: a repeat UK national survey. Arch Dis Child Fetal Neonatal Ed.

2017 Nov;102(6):F558. doi: 10.1136/archdischild-2017-313378. Epub 2017 Aug 5.

PMID: 28780498.

77: Oddie S, Stenson B, Wyllie J, Ewer AK. UK consultation on pulse oximetry

screening for critical congenital heart defects in newborns. Lancet. 2019 Jul

13;394(10193):103-104. doi: 10.1016/S0140-6736(19)31515-6. Epub 2019 Jul 1.

PMID: 31272689.

78: Cloete E, Gentles TL, Webster DR, Davidkova S, Dixon LA, Alsweiler JM,

Bloomfield FH; Pulse Oximetry Screening Steering Committee. Pulse oximetry

screening in a midwifery-led maternity setting with high antenatal detection of

congenital heart disease. Acta Paediatr. 2020 Jan;109(1):100-108. doi:

10.1111/apa.14934. Epub 2019 Aug 8. PMID: 31298757; PMCID: PMC6972617.

79: Hoff H, Quary S, Keesari R, Oster ME. Evaluating the Modified American

Academy of Pediatrics Screening Algorithm for Critical Congenital Heart Disease.

Am J Perinatol. 2025 Apr;42(5):674-682. doi: 10.1055/a-2416-5637. Epub 2024 Sep

17. PMID: 39288909.

80: Liberman RF, Heinke D, Lin AE, Nestoridi E, Jalali M, Markenson GR, Sekhavat

S, Yazdy MM. Trends in Delayed Diagnosis of Critical Congenital Heart Defects in

an Era of Enhanced Screening, 2004-2018. J Pediatr. 2023 Jun;257:113366. doi:

10.1016/j.jpeds.2023.02.012. Epub 2023 Feb 28. PMID: 36858148.

81: Taksøe-Vester CA, Mikolaj K, Petersen OBB, Vejlstrup NG, Christensen AN,

Feragen A, Nielsen M, Svendsen MBS, Tolsgaard MG. Role of artificial-

intelligence-assisted automated cardiac biometrics in prenatal screening for

coarctation of aorta. Ultrasound Obstet Gynecol. 2024 Jul;64(1):36-43. doi:

10.1002/uog.27608. Epub 2024 Jun 3. Erratum in: Ultrasound Obstet Gynecol. 2025

Apr;65(4):512. doi: 10.1002/uog.29156. PMID: 38339776.

82: Leone DM, Ittleman B, Virk K, Albright C, Arya B, Deen J. Screening for

Structural Heart Defects: A Single-Center Retrospective Cost Analysis for Fetal

Echocardiography in Adults with Congenital Heart Disease. Pediatr Cardiol. 2026

Jan;47(1):292-301. doi: 10.1007/s00246-024-03765-6. Epub 2025 Jan 15. PMID:

39812797.

83: Cloete E, Gentles TL, Alsweiler JM, Dixon LA, Webster DR, Rowe DL,

Bloomfield FH. Should New Zealand introduce nationwide pulse oximetry screening

for the detection of critical congenital heart disease in newborn infants? N Z

Med J. 2017 Jan 13;130(1448):64-69. PMID: 28081558.

84: Ismail AQT, Cawsey M, Ewer AK. Newborn pulse oximetry screening in practice.

Arch Dis Child Educ Pract Ed. 2017 Jun;102(3):155-161. doi:

10.1136/archdischild-2016-311047. Epub 2016 Aug 16. PMID: 27530240.

85: Miller R, Martens T, Jodhka U, Tran J, Lion R, Bock MJ. Effects of universal

critical CHD screening of neonates at a mid-sized California congenital cardiac

surgery centre. Cardiol Young. 2022 Feb;32(2):236-243. doi:

10.1017/S1047951121001797. Epub 2021 May 24. PMID: 34024296.

86: Fenster ME, Hokanson JS. Heart murmurs and echocardiography findings in the

normal newborn nursery. Congenit Heart Dis. 2018 Sep;13(5):771-775. doi:

10.1111/chd.12651. Epub 2018 Jul 24. PMID: 30039518.

87: Taivassalo K, Valkama M, Leskinen M, Ojaniemi M. Performing Pulse Oximetry

Screening in the Delivery Room Identified Newborn Infants Who Required Early

Medical Attention. Acta Paediatr. 2025 Nov;114(11):2976-2984. doi:

10.1111/apa.70202. Epub 2025 Jun 27. PMID: 40576441; PMCID: PMC12520277.1

88: Plana MN, Zamora J, Suresh G, Fernandez-Pineda L, Thangaratinam S, Ewer AK.

Pulse oximetry screening for critical congenital heart defects. Cochrane Database Syst Rev. 2018 Mar 1;3(3):CD011912.doi: 10.1002/14651858.CD011912.pub2.PMID: 40576441; PMCID: PMC12520277.1

89: Thangaratinam S, Brown K, Zamora J, Khan KS, Ewer AK. Pulse oximetry

screening for critical congenital heart defects in asymptomatic newborn babies:

a systematic review and meta-analysis. Lancet. 2012 Jun 30;379(9835):2459-2464.

doi: 10.1016/S0140-6736(12)60107-X. Epub 2012 May 2. PMID: 22554860.

90: Ewer AK, Middleton LJ, Furmston AT, Bhoyar A, Daniels JP, Thangaratinam S,

Deeks JJ, Khan KS; PulseOx Study Group. Pulse oximetry screening for congenital

heart defects in newborn infants (PulseOx): a test accuracy study. Lancet. 2011

Aug 27;378(9793):785-94. doi: 10.1016/S0140-6736(11)60753-8. Epub 2011 Aug 4.

PMID: 21820732.

91: Narayen IC, Blom NA, van Geloven N, Blankman EIM, van den Broek AJM, Bruijn

M, Clur SB, van den Dungen FA, Havers HM, van Laerhoven H, Mir SE, Muller MA,

Polak OM, Rammeloo LAJ, Ramnath G, van der Schoor SRD, van Kaam AH, Te Pas AB;

POLAR study group. Accuracy of Pulse Oximetry Screening for Critical Congenital

Heart Defects after Home Birth and Early Postnatal Discharge. J Pediatr. 2018

Jun;197:29-35.e1. doi: 10.1016/j.jpeds.2018.01.039. Epub 2018 Mar 23. PMID:

29580679.

92: Aranguren Bello HC, Londoño Trujillo D, Troncoso Moreno GA, Dominguez Torres

MT, Taborda Restrepo A, Fonseca A, Sandoval Reyes N, Chamorro CL, Dennis Verano

RJ. Oximetry and neonatal examination for the detection of critical congenital

heart disease: a systematic review and meta-analysis. F1000Res. 2019 Mar

1;8:242. doi: 10.12688/f1000research.17989.1. PMID: 31372214; PMCID: PMC6659768.

**Table 2.** ESC 2020 Classification of Congenital Heart Disease (Exam Table) (from Baumgartner et al. EHJ, 2020, citation 33)

| Complexity Level | Definition | Typical Lesions / Conditions |  |
| --- | --- | --- | --- |
| MILD | Simple, isolated defects with low long-term risk (if repaired or small) | • Small ASD, VSD, PDA • Repaired ASD/VSD/PDA (no sequelae) • Mild pulmonary stenosis • Isolated congenital valve disease (e.g. bicuspid aortic valve, mild mitral disease) |  |
| MODERATE | Intermediate complexity; may require intervention and lifelong surveillance | • AVSD (partial/complete) • Primum ASD • Moderate/large ASD • Coarctation of the aorta • Sub/supravalvular aortic stenosis • Anomalous coronary arteries (e.g. ALCAPA) • Partial/total anomalous pulmonary venous return • Ebstein anomaly (less severe) • Repaired Tetralogy of Fallot (uncomplicated) |  |
| SEVERE (GREAT COMPLEXITY) | Complex anatomy, high morbidity, lifelong specialized care required | • Cyanotic CHD (unrepaired/palliated) • Eisenmenger syndrome • Single ventricle / Fontan circulation • Transposition of great arteries (TGA) • Double outlet right ventricle (DORV) • Truncus arteriosus • Hypoplastic left heart syndrome • Pulmonary atresia ± VSD • Congenitally corrected TGA (ccTGA) • Complex Tetralogy of Fallot • Heterotaxy syndromes • Any CHD with severe complications (PAH, ventricular dysfunction) |  |

**Table 3 Online Supplementary Material.** Post Hoc Bayesian Estimation of diagnostic performance assuming a 95% sensitivity

Assumptions: 1000-patient cohort, 1% disease prevalence (10 disease cases), and 95% sensitivity.

| Specificity | False Positives | True Positives | False Negatives | Total Positive Tests | Probability a Positive is False |
| --- | --- | --- | --- | --- | --- |
| 0.90 | 99.0 | 9.5 | 0.5 | 108.5 | 91.2% |
| 0.91 | 89.1 | 9.5 | 0.5 | 98.6 | 90.4% |
| 0.92 | 79.2 | 9.5 | 0.5 | 88.7 | 89.3% |
| 0.93 | 69.3 | 9.5 | 0.5 | 78.8 | 87.9% |
| 0.94 | 59.4 | 9.5 | 0.5 | 68.9 | 86.2% |
| 0.95 | 49.5 | 9.5 | 0.5 | 59.0 | 83.9% |
| 0.96 | 39.6 | 9.5 | 0.5 | 49.1 | 80.7% |
| 0.97 | 29.7 | 9.5 | 0.5 | 39.2 | 75.8% |
| 0.98 | 19.8 | 9.5 | 0.5 | 29.3 | 67.6% |
| 0.99 | 9.9 | 9.5 | 0.5 | 19.4 | 51.0% |

Formula used: False Positives = (1 − specificity) × 990

True Positives = sensitivity × diseased patients = 0.95 × 10 = 9.5

**Online Supplementary Figure Caption.**

Cartoon reporting approximate location of acoustic probe location for all sound sampling. Details in the text.
