# Supplementary figures and images for: Rational and design of the digital diagnosis of cardiac sounds in paediatric patients (DI_SOUND) study
Source: Eur Heart J Digit Health. 2026 Jul 2;7(6):ztag103. doi: 10.1093/ehjdh/ztag103 (PMC13361984; doi:10.1093/ehjdh/ztag103)

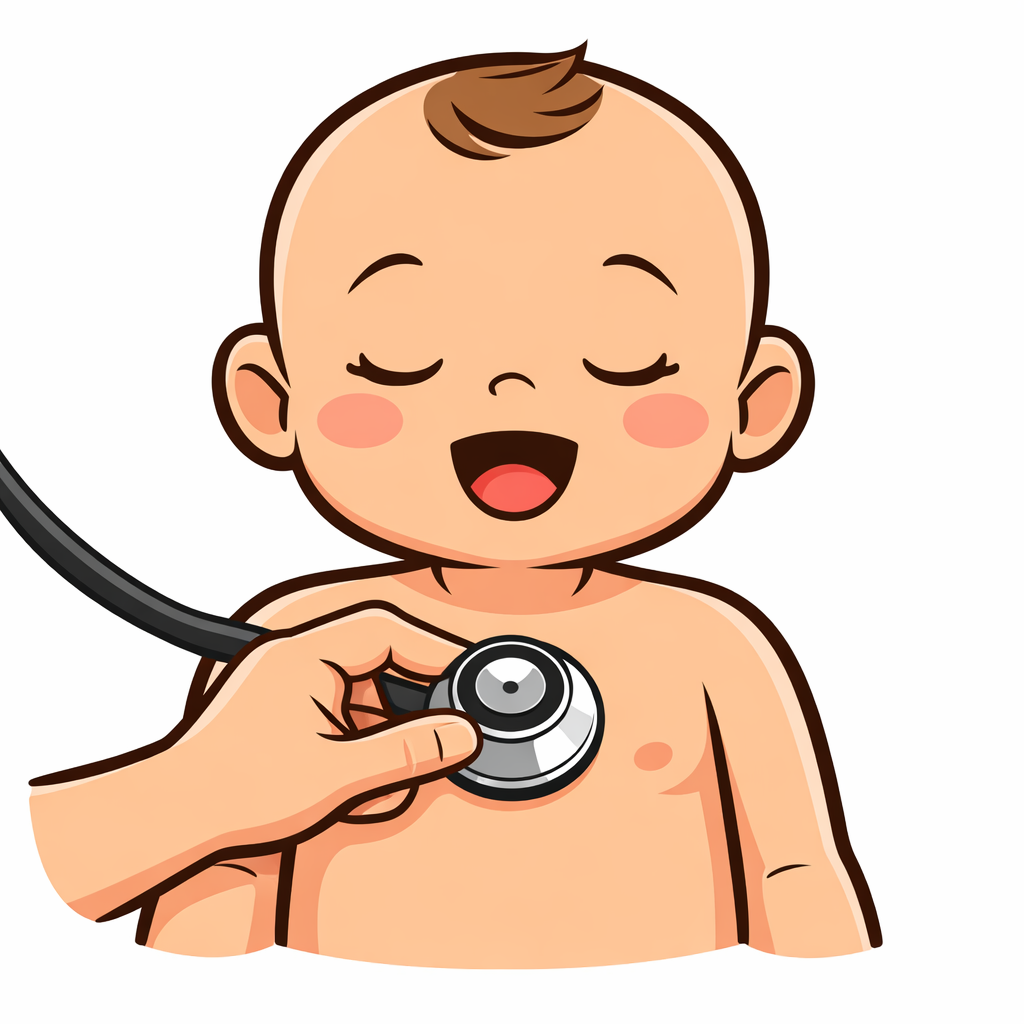

Supplement: ztag103_Supplementary_Data [file ztag103_supplementary_data.zip › DI_SOUND_Study_Design_Figure_Online_Supplementary_Material.png]
